# An Integrated Biorefinery Process to Revalorize Marine Biomass from the Microalga *Nannochloropsis gaditana* Using Pressurized Green Solvents

**DOI:** 10.3390/md23070263

**Published:** 2025-06-23

**Authors:** Cristina Blanco-Llamero, Paz García-García, Francisco Javier Señoráns

**Affiliations:** 1 Facultad de Ciencias de la Salud, Universidad Francisco de Vitoria, Ctra. Pozuelo-Majadahonda Km 1, 800, Pozuelo de Alarcón, 28223 Madrid, Spain; cristina.blanco@ufv.es; 2 Department of Food Science, Aarhus University, Aarhus Centrum, 8000 Aarhus, Denmark; 3 Healthy Lipids Group, Faculty of Sciences, Universidad Autónoma de Madrid, 28049 Madrid, Spain; javier.senorans@uam.es

**Keywords:** microalgae, *Nannochloropsis gaditana*, pressurized liquid extraction (PLE), biorefinery, carotenoids, byproducts

## Abstract

Biorefinery is gaining attention as a promising approach to valorize natural resources and promote a circular bioeconomy. This study aimed to recover high-value molecules, such as xanthophylls and polar lipids with nutraceutical applications, through enzymatic pretreatment and sequential pressurized liquid extraction (PLEseq), by reusing the residual biomass of *Nannochloropsis gaditana* after each processing step. Remarkably, pure glycolipids (102.95 ± 1.10 mg g^−1^ dry weight) were obtained immediately after enzymatic pretreatment, facilitating their easy recovery. Furthermore, two alternative sequential extraction processes were successfully developed, using ethanol and water as green solvents at varying temperatures and in different orders. The most effective PLEseq conditions yielded up to 48 mg mL^−1^ of carbohydrates using water at 50 °C, and up to 44 mg mL^−1^ of proteins via subcritical water extraction at 100 °C, prior to conventional lipid extraction with ethanol to produce various concentrated extracts. In the inverted PLEseq process—starting with ethanol extraction followed by successive water washes—isolated and purified fractions of lutein and astaxanthin were obtained, contributing to the complete depletion of the residual biomass. Overall, the development of an integrated and sequential biorefinery protocol that enables the extraction of multiple high-value compounds holds significant potential for application in the food industry.

## 1. Introduction

The wide range of bioactive compounds and molecules derived from microalgae makes these microorganisms a valuable bioresource with significant potential for exploitation in the food, pharmaceutical, and chemical industries. Microalgae are known to produce a diverse range of compounds including carotenoids, lipids, proteins, and carbohydrates, depending on the species involved. However, most extraction studies focus on a single target product, often disregarding the residual biomass and other potential co-products. Consequently, innovative industrial strategies have been proposed to enhance the production of microalgae-based products [1].

The chemical composition of microalgal biomass is highly promising for generating multiple products of interest across various sectors, including animal feed, food, fertilizers, fuels, and pharmaceuticals. Fully exploiting this potential requires a biorefinery approach that integrates pretreatment operations, thermochemical, biological, and catalytic processes, as well as separation techniques, to obtain both primary products and valuable co-products, thereby enabling the efficient use of bioresources [2,3,4,5].

Nevertheless, many aspects of microalgae processing remain exploratory due to the unique characteristics of each microalgal species. It is therefore essential to develop a comprehensive process from cultivation and harvesting to the extraction of high-value compounds. Some microalgae possess rigid cell walls that hinder the extraction of bioactive compounds; thus, a pretreatment step is often necessary to increase accessibility and maximize compound recovery in subsequent extraction steps. Additionally, after compound extraction, purification, concentration, and fractionation steps may be required to obtain purer and more highly concentrated extracts [6,7,8,9,10].

**Figure 1:**
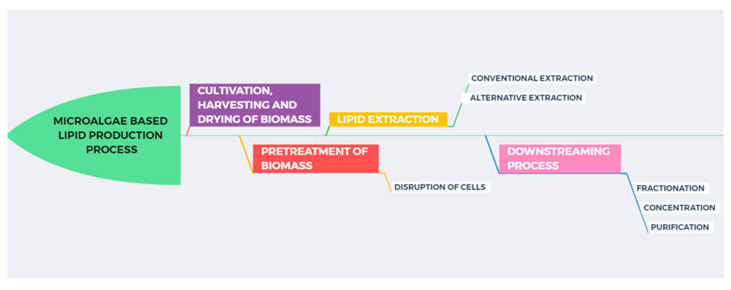
Microalgae-based lipid production diagram.

Figure 1 illustrates a microalgae-based lipid production diagram, highlighting all key steps required to obtain an optimal extract. The revalorization of co-products obtained throughout the production chain supports the circular bioeconomy and prevents the underutilization of biomass, which may still contain high-value compounds even after the initial extraction. In a biorefinery context, these co-products can often be obtained in concentrated fractions, minimizing the need for downstream processing. Beyond fuels and nutraceuticals, microalgal biomass is also being explored as a sustainable alternative to chemical fertilizers, contributing to carbon mitigation and improved soil health [11].

Recent innovations in low-energy harvesting and green extraction technologies, such as membrane filtration, supercritical CO_2_, and ionic liquids, are enhancing the efficiency and environmental sustainability of microalgae processing [12].

In recent years, *Nannochloropsis gaditana* has garnered increasing interest not only for its high lipid and carotenoid content but also for its potential as a sustainable feedstock in various industrial applications. Its bioactive compounds such as eicosapentaenoic acid (EPA), glycolipids, lutein, and astaxanthin have demonstrated antioxidant, anti-inflammatory, and cardiovascular benefits, supporting their use in nutraceuticals, functional foods, aquaculture feed, and cosmeceuticals [13,14].

The implementation of a biorefinery approach to valorize this microalga could significantly enhance the economic viability of microalgal biotechnology by generating multiple revenue streams from a single biomass input. Recent estimates suggest that global microalgal biomass production exceeds 19,000 tons annually, with *N. gaditana* being one of the most cultivated marine strains due to its rapid growth and high productivity under both phototrophic and heterotrophic conditions. Large-scale cultivation systems, such as raceway ponds and closed photobioreactors, have been successfully used for *N. gaditana*, particularly in Southern Europe and Asia, where favorable climate conditions enable cost-effective production. Consequently, integrating biorefinery strategies with *N. gaditana* biomass could play a pivotal role in scaling up sustainable marine biotechnology, reducing process waste, and promoting circular bioeconomy principles [13,14,15,16].

There is a growing emphasis on techno-economic analyses and environmental impact assessments to evaluate the feasibility of microalgae-based biorefineries. These tools are instrumental in identifying bottlenecks and optimizing process designs for industrial scalability [17].

Among the various microalgae species, *N. gaditana* stands out as a prolific lipid producer, rich in bioactive compounds with well-documented health benefits, such as omega-3 polyunsaturated fatty acids (PUFAs) like EPA and carotenoids including xanthophylls and β-carotene. Additionally, it contains proteins and carbohydrates of interest to the pharmaceutical and food industries. Lipid extraction from *N. gaditana* has been extensively studied using green techniques such as microwave-assisted extraction (MAE), ultrasound-assisted extraction (UAE), and pressurized liquid extraction (PLE) [18,19,20,21,22].

PLE is recognized as a versatile and automated technique that enables easy and scalable sequential extractions, facilitating the reuse of residual biomass after each extraction step [2,23,24,25]. However, due to its rigid cell wall, compound extraction from *N. gaditana* can be challenging, a problem that can be addressed by applying pretreatment methods to disrupt the cell wall. Several studies have investigated pretreatment strategies for *N. gaditana*, and a recent approach combining enzymatic and ultrasound treatments has been shown to triple the oil yield in subsequent extraction steps [26,27,28,29].

Recent research highlights the development of multiproduct biorefineries that integrate biofuel production with the extraction of high-value compounds (e.g., pigments, proteins, and bioactives). These models aim to enhance both economic viability and sustainability by valorizing all biomass fractions. Indeed, the concept of the circular bioeconomy is increasingly embedded in microalgal biorefinery strategies, with added emphasis on CO_2_ capture, wastewater treatment, and nutrient recycling. Growing policy support and patent activity further reflect a maturing and evolving field.

Thus, the present work aims to evaluate whether green technologies, such as enzymatic pretreatment and PLE using environmentally friendly solvents, can selectively extract and fractionate high-value compounds from microalgal biomass. The goal is to develop a sustainable and eco-friendly process aligned with the United Nations Sustainable Development Goals.

## 2. Results

### 2.1. Enzymatic Pretreatment Byproducts—Direct Enzymatic-Assisted Extraction (EAE)

According to the results obtained in the optimization of the ultrasound-assisted protocol in previous works [26], in this article, we combined three different commercial enzymes under the optimal conditions (55 °C and pH 5.0 during 6 h) in order to prove their efficiency to improve the total oil yield.

On one hand, the supernatant after the pretreatment, which is usually discarded in pretreatment works, was evaporated under nitrogen steam and was analyzed as well to compare both fractions, the supernatant and the extract from the pretreated biomass. Interestingly, High-Performance Liquid Chromatography (HPLC) analysis revealed that the supernatant also contained lipids; specifically, it contained pure glycolipids (GL, Table 1). These results showed that direct enzymatic-assisted extraction of the biomass was also occurring during the application of the pretreatment. Thus, a portion of the glycolipids was extracted and released into the medium without requiring an additional step due to the affinity for polar media (in this case, buffer). This way, instead of obtaining one extract, the developed process obtained two valuable extracts of lipids without adding any extra step nor employing more solvents or energy, as a green alternative from a biorefinery approach.

Additionally, fatty acid analysis was also performed using Gas Chromatography–Mass Spectrometry (GC-MS) with promising results, as shown in Table 1. The content of lipids in the PLE extract was much higher than that in the EAE, as was expected. However, it could not be considered a depreciable fraction as it contains more than half of the glycolipids presented in the PLE extract, and it was purer as it was concentrated at 97%, containing 28% of EPA among its fatty acid (FA) composition. This is the reason why these results found a new highly valuable fraction of *N. gaditana*, integrated in the pretreatment method, which used to be discarded and could have a promising application in the pharmaceutical and food industry.

### 2.2. Sequential PLE to Revalorize Marine Products from N. gaditana Biomass

#### 2.2.1. First Approach: Isolation of High-Added-Value Molecules

The choice of solvent plays a crucial role in the selectivity of the extracted compounds. In this study, the use of water allowed efficient extraction of carbohydrates and proteins at subcritical temperatures, while ethanol favored the recovery of lipids and carotenoids. The polarity of the solvent, along with the applied temperature, directly influences the solubility and mass transfer of bioactive compounds. PLE for *N. gaditana* was previously optimized in recent works of the group in terms of lipidic and carotenoid extracts [27], obtaining optimum conditions with ethanol and 120 °C. However, microalgae have been described to contain a wide range of valuable compounds, not only lipids. Consequently, a sequential extraction process to exhaust microalgal biomass was required; thus, carotenoid, carbohydrate, and protein extraction was investigated employing water as the extracting solvent and different temperatures to achieve a greener process.

As a first stage, carbohydrate extraction was investigated using sequential PLE. Three temperatures under subcritical points were investigated (25, 50, and 75 °C), and then, the extracts were analyzed in terms of carbohydrates, protein, and carotenoid content by spectrophotometric methods.

**Figure 2:**
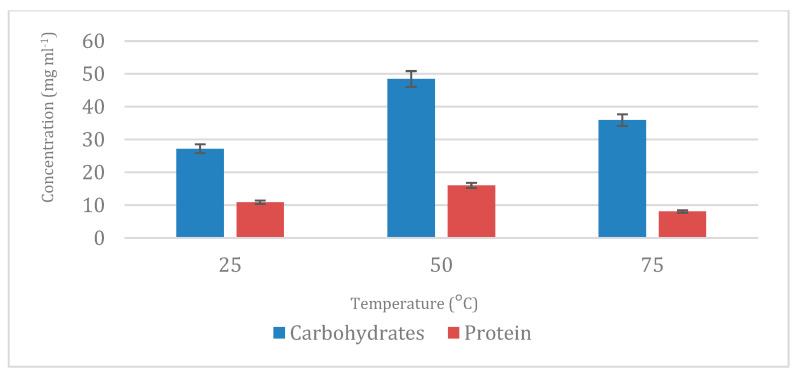
Total protein and carbohydrate content extracted and analyzed in step 1 at different temperatures using water as green solvent (25, 50, and 75 °C).

Although carbohydrates and proteins are generally poorly soluble in ethanol, detectable amounts were observed in the ethanolic extracts. This can be explained by co-extraction phenomena, partial denaturation of proteins that increase their affinity for less polar solvents, or the presence of water-soluble compounds partially soluble in ethanol due to their chemical structure. Interestingly, once the PLE was applied and the extracts were analyzed, the results of carbohydrates widely ranged from 25 to 48 mg mL^−1^, whereas the protein content varied from 8.58 to 14.93 mg mL^−1^ depending on the conditions tested. The largest percentage of carbohydrates was obtained using 50 °C (Figure 2). The efficiency of water at 50 °C in extracting carbohydrates can be attributed to the partial rupture of the cell wall induced by enzymatic pretreatment, facilitating the release of soluble polysaccharides. Likewise, obtaining pure glycolipids after pretreatment represents a significant advantage, as it avoids additional purification steps and reduces solvent use.

**Figure 3:**
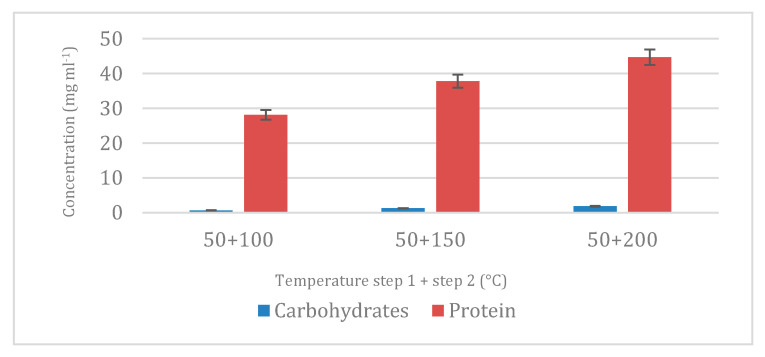
Total protein and carbohydrate content extracted and analyzed in step 2 at different temperatures (100, 150, and 200 °C) using water as green solvent. In all cases, the first step was developed at 50 °C.

At a second stage, proteins were extracted employing subcritical conditions with water (SWE) with temperatures of 100, 150 and 200 °C. As can be seen in Figure 3, a major protein percentage was achieved as the temperature increased as expected, due to a major transference of material with high temperatures, whereas the content of carbohydrates in this stage was much lower than in the first stage, meaning that most of the carbohydrates presented in *N. gaditana* was successfully extracted with the previous extraction step, obtaining an extract rich in proteins.

**Figure 4:**
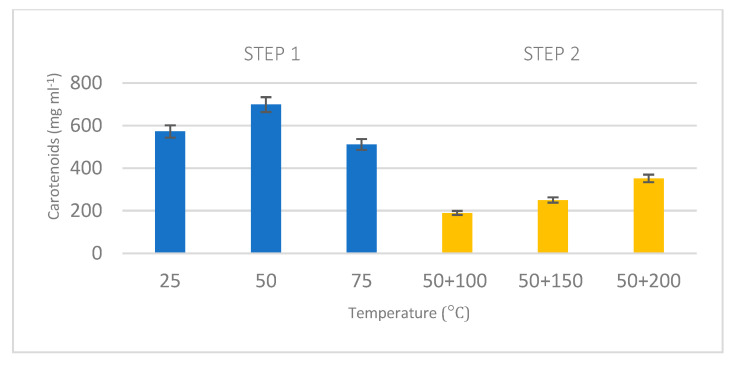
Comparison between total carotenoid content extracted in step 1 and steps 1 + 2. The first extraction was developed using water at different temperatures (25, 50, and 75 °C). Second sequential extraction was developed using water at 100, 150, and 200 °C.

On the other hand, optimum temperatures for carbohydrates and proteins were in agreement with best conditions for carotenoid extraction, as shown in Figure 4, where most of the carbohydrates was extracted in the first stage with 50 °C and its residual content was extracted then in the second step at 200 °C.

Once the different temperatures were investigated, the whole process was applied adding the third step of lipidic extraction as previously optimized in another work by the research group [27]. Thus, the process consisted of a first phase (P1) of PLE with water at 50 °C to extract carbohydrates, a second phase (P2) of PLE with water at 200 °C to extract proteins, and a third phase (P3) of PLE with ethanol at 120 °C. The results of the composition of the third extract are shown in Table 2.

When using higher temperatures in the second step of the sequential process, the extract had a lower amount of EPA compared with the similar single-step process (P3) in which ethanol was used. Thus, 100 °C was chosen instead of 200 °C to integrate the whole process, and to preserve a third extract rich in lipids and carotenoids, as the composition of the other ingredients in the third extract was similar either using one temperature or another.

Consequently, as it has been shown, the steps applied before the lipidic extraction resulted in three different extracts, one per stage, the first one with the highest content of carbohydrates and carotenoids, the second one with a major percentage of proteins, and the third one with almost nonexistent carbohydrates or proteins (Table 2). However, the third extract also did not have a high amount of carotenoids compared to the extract without the previous steps of sequential extraction (see P3 in Table 2), as they were previously mostly extracted in stage 1 and 2. It is important to highlight the amount of carotenoids extracted in each process with each temperature, either 100 or 200 °C. The third extract using 200 °C in the second step contained a remarkably lower amount of carotenoids as they had been partially extracted in the previous step of protein extraction, obtaining higher amounts when lower temperatures were used in the second steps.

#### 2.2.2. Second Approach: Depletion of Biomass

The sequential process allows an orderly fractionation of carbohydrates, proteins, and lipids, while the inverted process prioritizes the preservation of sensitive lipids and carotenoids. The choice of approach depends on the final goal: maximum recovery of specific compounds or integral utilization of biomass. The inversion of the process was performed using a first step of PLE with ethanol at 120 °C, followed by a second step of water at 50 °C, and a third one of water at 100 °C, regarding the conditions obtained in the first approach. Then, two extra washes with water at the highest temperature employed (120 °C) were enough to exhaust the microalgal biomass, performing lipid extraction in the first place instead of the last to preserve sensitive compounds such as PUFAs.

**Figure 5:**
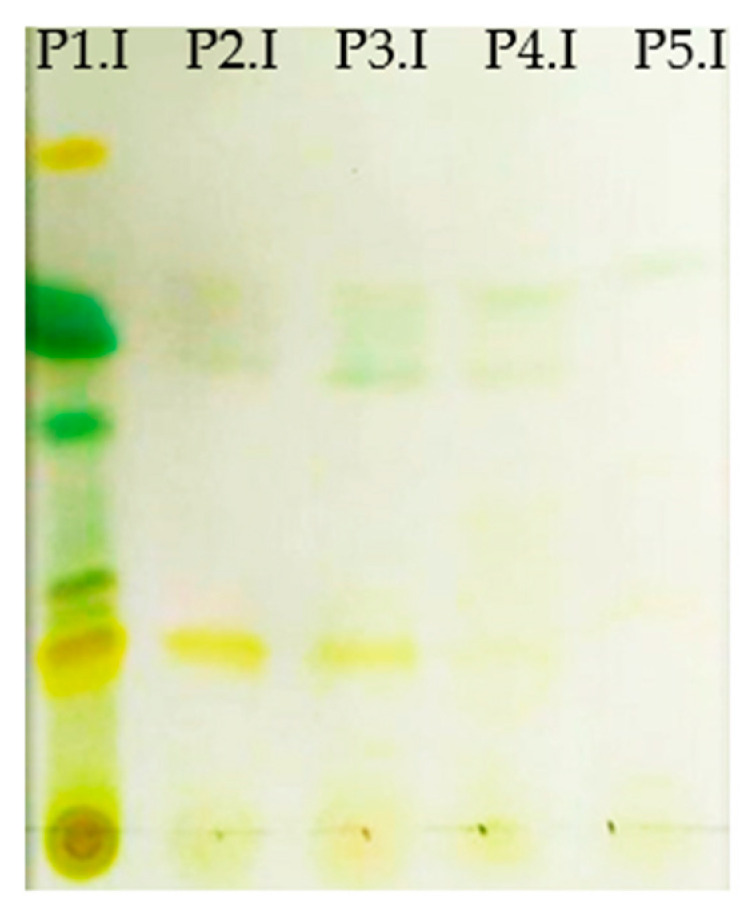
Carotenoid identification and separation of the PLE extracts (from P1.I on the **left** side to P5.I on the **right** side) with the inverted process using TLC Silica plates. P1.I corresponded to phase 1 of the inverted process (PLE ethanol), P2.I to phase 2 (PLE water) and P3.I to phase 3 (PLE water). P4.I and P5.I correspond with two additional washes with water.

As it can be seen in Figure 5, depletion of the biomass in terms of carotenoid extraction was obtained with sequential steps in the inverted process of sequential PLE. Sequential extractions enabled the obtention of purified fractions enriched in carotenoids (from P2 to P5) that could have remained in the biomass if conventional extraction with ethanol would be used.

On the other hand, this system allowed us to obtain fractions of carotenoids in each phase with minimal amounts of carbohydrates and proteins, as they were completely extracted in the first stage (see P1.I in Table 3).

As seen in Table 4, the carotenoids in the different fractions obtained were analyzed determining the first phase as a rich extract of lipids and a wide range of carotenoids as β-carotene, astaxanthin, lutein, and chlorophylls. However, the results revealed that the extracted biomass still contained valuable products. The second phase contained pure lutein and residual amounts of chlorophylls, whereas the third one contained lower amounts of lutein and chlorophylls, as they were first extracted, but higher amounts of astaxanthin that increased with the sequential extractions. Additionally, β-carotene was also found in the first extract, obtaining a fractionated range of carotenoids in the sequential phases. Finally, to confirm that the biomass was totally exploited, two extra washes with water at 120 °C were applied obtaining phase 4 and 5 and showing residual amounts of lutein until it disappeared.

## 3. Discussion

The results of two different approaches to valorize and deplete the harsh biomass of *N. gaditana* are discussed here.

Due to the presence of a dense and firm microalgae cell wall, especially for *N. gaditana* where the cell wall is a bilayer structure protected by an external hydrophobic algaenan layer, extracting lipids from microalgae becomes a difficult task. For this reason, microalgae cell wall must be properly disrupted to efficiently enhance lipid recovery. Different methods for cell disruption are based on mechanical, chemical, physical, and biological approaches and have been described in the literature. Among these, enzymatic cell wall disruption is considered an environmentally friendly alternative method with several advantages such as mild reaction conditions, higher selectivity, and less energy consumption than other pretreatment methods [1,2,3,4].

In this work, after the application of enzymes under ultrasound action, the resulting biomass was extracted using green extraction techniques such as PLE, as it was optimized in previous works, testing different temperatures and solvents to effectively extract different types of compounds with different applications in industry [30,31]. Previous studies in our laboratory demonstrated that PLE can use a wide range of solvents, including green solvents such as ethanol or water and mixtures of them to easily extract polar lipids [27], and can achieve results similar to those of traditional organic solvents in a greener way.

**Figure 6:**
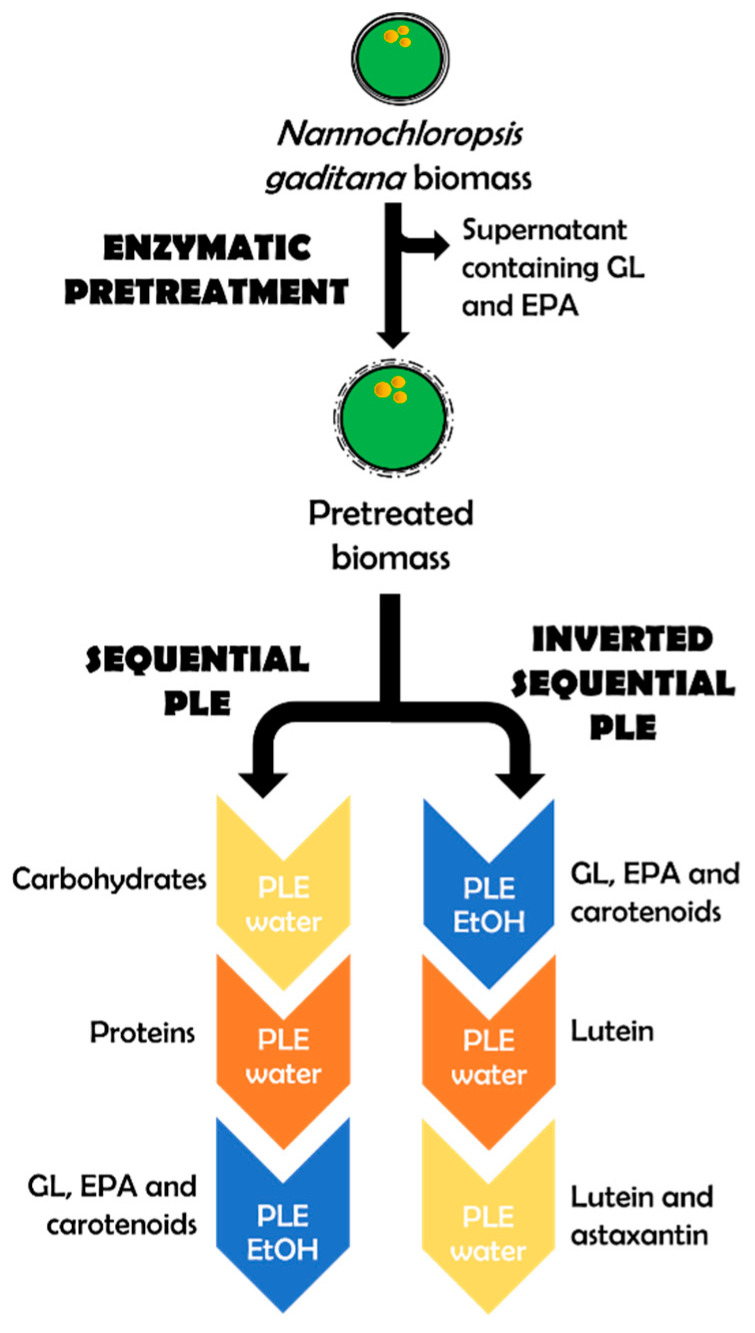
Schematic representation of the whole process developed where different valuable fractions of *N. gaditana* were obtained after enzymatic pretreatment. Sequential PLE and inverted sequential PLE are represented.

Thus, the versatility of PLE enabled developing a new sequential extraction method for simultaneous fractionation on the microalga biomass combining different green solvents and extraction conditions to revalorize marine products that were previously reported [7,30]. The amount of bioactive compounds with high-added value such as lipids, carotenoids, proteins, and carbohydrates was evaluated among these two approaches performed in inverted order (Figure 6).

On one hand, preliminary works on PLE [20,22] reported that low temperatures under supercritical conditions with water as the extracting solvent were enough to extract carbohydrates under pressure. The largest percentage of carbohydrates obtained in this work was using 50 °C, which is in agreement with previous works on carbohydrate extraction using PLE and water that achieved its highest results at the same conditions [24,32]. Once the first step of carbohydrates was developed and the optimum temperature was chosen, a second stage of water extraction was performed at more aggressive conditions to extract proteins from microalgae, and the results were again in accordance with previous results in which proteins were extracted at 100 °C using SWE. Interestingly, the detection of carbohydrates and proteins in ethanol-based extracts (P1.I) may be attributed to a combination of factors, including partial hydrolysis of cellular structures during enzymatic pretreatment and the influence of pressurized conditions that alter solvent polarity and solute interactions. Although ethanol is typically associated with the extraction of non-polar or moderately polar compounds, studies have shown that at elevated temperatures and pressures, it can solubilize a wider range of compounds due to decreased dielectric constant and increased diffusivity. This could explain the recovery of non-lipid compounds in ethanol fractions, especially following cell wall disruption by enzymes and ultrasound. Such findings suggest that solvent performance under pressurized conditions may differ significantly from conventional expectations, reinforcing the importance of tuning extraction conditions for selectivity [33,34]. Regarding carotenoid extraction, carotenoids were extracted when high temperatures were used, and the content improved as the process raised the operational temperature [8].

On the other hand, regarding the extraction using ethanol to effectively extract lipids, the sequential extraction after a 200 °C step to recover proteins seemed to be decisive for EPA. In this way, the extract had lower amount of EPA compared with the similar one-step process in which ethanol is used, which could be directly attributed to the highest temperatures tested in the second step that may damage PUFAs and sensitive compounds in the biomass as carotenoids. Indeed, previous works of the research group demonstrated that temperatures up to 150 °C produced lipid oxidation increasing the amount of monoglycerides and free fatty acids in the extracts [20,22,27].

This study introduces several relevant innovations in the field of microalgae biorefinery. First, the pure glycolipids obtained directly from enzymatic pretreatment without subsequent extraction are demonstrated for the first time. Second, two sequential extraction strategies (direct and inverted) are compared, allowing the process to be adapted according to the industrial objective (preservation of lipids vs. complete fractionation).

Thus, the inverted process was also intended to explode microalgal biomass after lipidic extraction as a different alternative process to preserve an extract rich in lipids and carotenoids altogether. Consequently, two alternative processes may be useful for different objectives, the first one with sequential extraction of different compounds separately, and the inverted process to reuse microalgal biomass and obtain added-value sub-products after lipid extraction, mainly isolated carotenoids.

Taking into consideration the amount of carotenoids obtained in the inverted process, these included a wide range of carotenoids including the more polar and non-polar ones with different applications, such as β-carotene, astaxanthin, lutein and chlorophylls. This can be attributed to the higher temperatures applied at this stage (100 °C) and to the fact showed by other works where the astaxanthin in microalgae are often bounded to complex proteins, which make it difficult to extract [35]. The sequential application of pressure to the biomass may contribute to the self-pretreatment facilitating the extraction of certain compounds, as different authors in the application of sequential PLE have argued [36,37,38,39]. This fact along with the high temperature applied in this step may explain the results obtained. These results support the use of the integrated method as a biorefinery process to reuse microalgal biomass and obtain valuable compounds from it after lipid extraction.

From a practical standpoint, the proposed process demonstrates several characteristics that facilitate industrial scale-up. Pressurized liquid extraction (PLE) systems are commercially available and scalable, and the use of GRAS solvents such as ethanol and water ensures regulatory compliance for food and nutraceutical applications. Enzymatic pretreatment, although cost-sensitive, can be optimized through enzyme immobilization or reuse strategies, which are currently under investigation in industrial settings. Moreover, by integrating multiple extraction steps from a single biomass input, the process maximizes product recovery while minimizing waste and processing time. Preliminary cost estimations from similar biorefinery setups suggest that coupling enzyme-assisted PLE with sequential valorization could reduce overall production costs by up to 25–35% compared to single-product processes, while increasing market competitiveness through diversified product portfolios.

This work brings several novel contributions to the field of algal biorefinery. First, it demonstrates for the first time the selective recovery of pure glycolipids during enzymatic pretreatment without the need for organic solvents, revealing a previously discarded high-value fraction. Second, it compares two novel sequential extraction strategies (direct and inverted PLE), offering versatile tools depending on the target compounds, and enabling complete biomass depletion. Third, it provides a scalable, solvent-efficient method using only ethanol and water under pressurized conditions to obtain fractionated extracts rich in EPA, lutein, and astaxanthin. To our knowledge, this is the first report that integrates enzyme-assisted extraction, sequential PLE, and inverted PLE in a unified, sustainable workflow applied to *N. gaditana*, with demonstrated potential for circular bioeconomy applications.

In summary, the proposed integrated biorefinery approach successfully optimized the sequential extraction of bioactive compounds from *N. gaditana*. The first extraction stage yielded carbohydrate-rich fractions at mild temperatures (50 °C), while subsequent steps at higher temperatures allowed for efficient protein recovery and lipid extraction, with ethanol proving to be a suitable green solvent. The reversed process demonstrated versatility, producing lipid- and carotenoid-rich fractions while enabling complete biomass utilization. These findings emphasize the potential of combining enzymatic pretreatment and pressurized liquid extraction to achieve a sustainable, scalable, and environmentally friendly valorization process, aligning with circular bioeconomy principles.

From an industrial perspective, the proposed process presents significant advantages. Pressurized liquid extraction (PLE) technology is already available at pilot and industrial scales, facilitating its implementation. The use of green solvents such as water and ethanol reduces operational costs and environmental risks compared to traditional organic solvents. Additionally, the sequential approach allows for obtaining concentrated extracts, minimizing the need for subsequent concentration or purification stages, thus reducing energy consumption. However, potential bottlenecks such as the availability and cost of commercial enzymes, energy consumption associated with pressure and temperature, and efficient solvent recovery must be considered [17].

Unlike previous studies focused on a single product, this work proposes an integral approach that maximizes biomass valorization. Furthermore, the transversal applicability of the method to other microalgae species and marine biomass is proposed, expanding its potential implementation in different industrial contexts.

Future research will be focused on the application of this promising method to other microalgal biomass containing different amounts of bioactive compounds, as well as seaweeds in which this type of extraction could enhance the yields and application of edible biomass.

## 4. Materials and Methods

### 4.1. Chemicals and Reagents

*N. gaditana* dry biomass was provided by Algaenergy S.A. (Alcobendas, Spain). Methanol was purchased from Labscan Analytical Sciences (Gliwice, Poland). Hexane and HPLC-grade solvents (2,2,4-trimethyl pentane, methyl tert-butyl ether (MTBE)) were purchased from Macron Fine Chemicals (Gliwice, Poland). Absolute ethanol (PRS grade), sodium hydrogen carbonate and potassium hydroxide were purchased from Panreac Química S.A (Barcelona, Spain). The water used was Milli-Q grade (Millipore Sigma, Burlington, MA, USA). Viscozyme^®^ from *Aspergillus aculeatus* containing a wide range of carbohydrases, including arabinase, cellulase, beta-glucanase, hemicellulase and xylanase, and Celluclast^®^ containing cellulase from *Trichoderma reesei* and Alcalase^®^ were kindly donated by Novozymes (Bagsvaerd, Denmark). Glyceryl trilinoleate, dioleoylglycerol (mixture of 1,3- and 1,2-isomers), 1-oleoyl-rac-glycerol, oleic acid and ethyl linoleate used as HPLC standards were purchased from Sigma Aldrich (St. Louis, MO, USA). All other reagents and solvents used were of analytical or HPLC grade. Fatty acid methyl esters standard (Supelco 37 FAME Mix) was from Supelco (Bellefonte, PA, USA).

### 4.2. Pretreatment Method Conditions

A total of 1 gram of dry microalgal biomass was resuspended in 10 mL of sodium citrate buffer 0.1M with pH 5.0 (ratio biomass: solvent 1:10) containing 46 mg of different enzymes (Viscozyme^®^, Alcalase^®^, and Celluclast^®^) (equally added up to 46 mg) per gram of biomass, and incubated under optimal conditions at 55 °C, according to [26]. The flask content was centrifuged at 3000 rpm for 10 min, and supernatant and pellet biomass was separated and kept at 4 °C for extraction and characterization of both using HPLC-ELSD and GC-MS.

### 4.3. HPLC-DAD/ELSD Analysis

HPLC with Evaporative Light Scattering Detector (HPLC-ELSD) analyses were performed using an Agilent 1260 Infinity HPLC equipped with an Agilent 385 (Palo Alto, CA, USA) ELSD instrument. The chromatographic separation of the different species of lipids (neutral and polar lipids) was performed with a silica normal-phase ACE (250 mm × 4.6 mm i.d. 0.5 µm) column maintained at 30 °C using a ternary gradient as follows: 0–3 min, 95% B and 5% C, 50% B and 50% C at *t* = 3 min; 2% A, 48% B and 50% C at *t* = 9 min; 60% A and 5% B and 35% C at *t* = 17 min; 75% A and 5% B and 20% C at *t* = 21 min; 50% B and 50% C at *t* = 31 min; and 95% B and 5% C at 33 min. Eluent A consisted of methanol, eluent B consisted of 2,2,4-trimethylpentane, and eluent C consisted of methyl tert-butyl ether. The flow rate was variable (1.0 or 2.0 mL/min) and programmed. The optimal signal and resolution were achieved with the following ELSD conditions: evaporator temperature = 30 °C; nebulizer temperature = 30 °C; and evaporator gas N2 = 1.6 SLM. Lipid species were identified using commercial standards. Results were expressed as the individual relative percentage of each lipid species present in the sample (normalized areas).

The composition of carotenoids in the extracts was analyzed by HPLC coupled with a photodiode array detector, carried out in a reverse phase C18 column. The mobile phase was a mixture of solvent A (water: methanol; 1:5) and solvent B (methanol: isopropanol; 3:1) according to a step gradient, lasting 33 min, starting from 20% B changing to 50% at 4 min, then raising up to 70 at 8 min and to 95% at 11 min. Finally, it changed to 99% B at 22 min, and the mobile phase composition was kept constant until the end of the analysis. The injection volume of the standards and the samples was 20 μL. The UV-spectra were obtained between 400 and 600 nm. The identification of the peaks was performed, when possible, using standards (astaxanthin, lutein and β-carotene). When no standards were available, tentative identification was based on retention time, UV-VIS spectral characteristics, and comparison with data available in the literature. To delve deeper into the results and to make possible comparison among different extracts, HPLC was used to quantify major peaks in the chromatograms. The quantification was performed using an external standard of lutein and astaxanthin. The linear regression equation for the standard curve was obtained by plotting the amount of standard compound injected against the peak area.

### 4.4. Fatty Acid Composition by GC-MS

Fatty acid composition of the obtained extracts was analyzed by GC–MS. Prior to analysis on an Agilent GC-MS series 5975 MSD (Palo Alto, CA, USA), fatty acid methyl esters (FAMEs) were freshly prepared by base-catalyzed methanolysis of the glycerides (KOH in methanol). FAMEs were separated using a HP 88 capillary column (100 m × 0.25 mm, i.d. 0.2 µm) (Agilent, Santa Clara, CA, USA). One µL sample was injected using a split ratio of 1:100. The column was held at 175 °C for 10 min after injection, the temperature programmed at 3 °C/min to 220 °C and maintained for 20 more minutes. Helium was used as a carrier gas, at a constant column flow rate of 1.5 mL/min. Injector temperature was 250 °C and the detector temperature was 230 °C. The mass spectrometer was operated at 70 eV with a mass range from 30 to 400 amu. Fatty acid methyl esters were identified by comparing their retention times and mass spectra (NIST MassSpectral Library Version 2.0) with those obtained from the standards. Results were expressed as the individual relative percentage of each fatty acid over all FAMEs in each sample.

### 4.5. Pressurized Liquid Extraction

All extractions were carried out in an accelerated solvent extraction system ASE 350 DIONEX extractor (Sunnyvale, CA, USA) equipped with stainless steel extraction cells (10 mL volume). Extractions were performed using ethanol or water as solvents at different extraction temperatures, according to the biorefinery design. Microalgal biomass was weighed (equivalent to 1 g dry biomass), mixed with sea sand (ratio 1:10), and loaded into the extraction cell. Then, the extraction cell was filled with the different solvents used and heated to the selected temperature (25–120 °C). The pressure was maintained at 1500 psi. The solvents used were ethanol, or pure water. Static extraction time was 15 min for each experiment and the solvent volume used was 20–25 mL, depending on the temperature and pressure of each extraction. Samples were stored at −20 °C to prevent degradation until analysis.

### 4.6. Sequential Process for the Downstream Valorization of Fractions from N. gaditana

A sequential process was developed for the valorization of bioactives. The first step consisted of the optimization of carbohydrates from the raw microalgal biomass using PLE for 20 min, using pure water at different temperatures (from 50 to 75 °C). After obtaining the optimum conditions for the recovery of carbohydrates, a second step on the residual biomass was studied using higher temperatures (100–200 °C) for the recovery of proteins. Once the two steps were optimized, lipid extraction was applied at the last step at 120 °C with ethanol, as it was previously optimized and reported in the literature [27]. Therefore, the integrated process was performed in three sequential steps using pure water at different temperatures (25–200 °C), and pure ethanol at 120 °C. Carotenoids and carbohydrates extraction was optimized using low temperatures (25–75 °C); then, temperature extraction was evaluated for protein and residual carotenoids extraction (100–200 °C). This way, three valuable isolated fractions were obtained from the microalgae biomass.

Once all conditions were optimized, and the whole process was developed. The inversion of the method was intended to obtain different fractions; Step 1: lipid extraction of *N. gaditana* biomass was performed as it was mentioned in previous papers [29]; Step 2: the residual biomass from the previous extraction was extracted again using PLE for 20 min, using pure water at 50 °C to extract residual carotenoids and carbohydrates; Step 3: the extraction was performed at the optimum extraction conditions for protein extraction using PLE for 20 min at 100 °C. Then, the residual biomass was exhausted using two extra washes of water at the highest temperature (120 °C).

### 4.7. Total Carotenoids Determination

A simple spectrophotometric method was used to determine the total carotenoids and total chlorophylls concentration, based on their characteristic absorbance. Extracts obtained during optimization were dissolved in methanol at a concentration of 0.25−1 mg mL^−1^. The absorbance of these solutions was recorded at a specific wavelength (470 nm) for total carotenoids. An external standard calibration curve of lutein (0.2–20 μg mL^−1^) was used to calculate the total carotenoids content [24].

### 4.8. Determination of Carbohydrates

Total carbohydrates content of the extracts obtained using water as solvent was determined using the phenol-sulfuric acid method. Briefly, 167 μL 5% phenol was mixed with 278 μL of extract (diluted in Milli-Q water at known concentration). Then, 1 mL sulfuric acid was added and carefully mixed. After 30 min at room temperature, absorbance was recorded at 490 nm. A standard calibration curve using glucose (0.03–1.0 mg mL^−1^) was used to determine the carbohydrates content [24].

### 4.9. Bradford Method for Protein Quantification

The protein concentration was determined by the method of Bradford [40]. The samples were diluted (1/2, 1/5, 1/10, 1/20) to obtain different enzymatic solutions. To perform the measurements, 20 μL of sample were added to 1 mL of Bradford solution and allowed to react for 30 min. The absorbance was measured at 595 nm on a model UV-Vis UV-1280 spectrophotometer. The absorbance range of the samples must be between 0.1 and 1 for measurements to be reliable. Different concentrations were obtained from a known standard curve for serum albumin bovine (BSA).

### 4.10. Analytical Thin-Layer Chromatography of the Samples Obtained for Carotenoid Identification

Thin-layer chromatography (TLC) was used to analyze carotenoids. A chromatographic silica plate was cut, and 1 cm (approx.) of mobile phase was added to the chromatographic chamber (Petroleum ether phase: acetone, 75:25). Then, 10 μL of the dissolved extracts of the different solvents was added together with a known Rf marker standard (β-carotene, 500 ppm concentration in isopropanol). Samples must be above the mobile phase line. The mobile phase was allowed to elute until the solvent front reaches ~1 cm from the edge of the plate (approximately 20 min).

### 4.11. Statistical Analysis

The results were expressed as the mean of the experiments and its standard deviation. Statistical analysis was performed in the SISA (Simple Interactive Statistical Analysis) online software available at [41]. The data were subjected to a *t*-test to examine whether two groups’ means differ from one another. To test if there is an overall statistically significant difference between three or more means, the data were subjected to a one-way analysis of variance (ANOVA) using the F test for discrimination between means. 

## 5. Conclusions

The present work demonstrates the significant potential of downstream processing strategies for microalgae biomass, highlighting the importance of revalorizing fractions that are often underestimated or discarded in conventional pretreatment and extraction stages. Our integrated biorefinery approach, combining enzymatic pretreatment with sequential pressurized liquid extraction (PLEseq), enables the efficient recovery of multiple high-value compounds from *N. gaditana* biomass, thereby maximizing resource utilization.

A key innovation of this study lies in the enzymatic pretreatment step, which not only facilitates the production of ready-to-extract biomass but also allows for the direct extraction of pure glycolipids, effectively yielding two valuable products simultaneously. This green and versatile pretreatment process simplifies downstream operations and enhances overall extraction efficiency. Moreover, the application of two alternative sequential PLE methods using green solvents (ethanol and water) demonstrates the versatility of the method as well as the scalability. Depending on the extraction sequence (inverted or conventional), it is possible to selectively recover concentrated fractions of xanthophylls such as lutein and astaxanthin, polar lipids, and carbohydrates and proteins, all from the same residual biomass. This sequential extraction not only increases the yield of valuable bioactive compounds but also minimizes the need for further concentration steps, saving both time and energy.

Thus, sequential PLE emerges as an automated and adaptable technology that readily couples sequential extractions in an efficient manner, suitable for industrial scalability. Together with the enzymatic pretreatment, this integrated protocol exemplifies a sustainable, circular bioeconomy-aligned process grounded in the principles of green chemistry and biorefinery using also sustainable and alternative biomass. In conclusion, the development of this biorefinery strategy offers a promising solution for the sustainable production of multiple high-value compounds from microalgae biomass. It supports enhanced economic viability, reduces process waste, and contributes to advancing sustainable practices within the food and nutraceutical industries.

## Figures and Tables

**Table 1:** Chemical composition of the extracts obtained from *N. gaditana*.

	GL (%) *	GL (mg g^−1^ dw)	EPA (%) *
EAE extract	97.23 ± 0.88	102.95 ± 1.10	28.55 ± 0.33
PLE extract	44.11 ± 1.01	186.11 ± 0.79	35.13 ± 0.48

* Results expressed as percentage over the total content (relative content). Values are the mean ± SD of two determinations.

**Table 2:** Chemical composition of the extracts obtained by the sequential process at different phase 2 temperatures (100 or 200 °C) compared to the PLE extraction without the sequential process applying only ethanol at 120 °C.

Phase	Temperature (°C)	Carbohydrates (mg mL^−1^)	Proteins (mg mL^−1^)	Carotenoids (mg mL^−1^)	GL (%) *	EPA (%) *
P1 + P2 + P3	50 + 100 + 120	10.96 ± 0.99	1.63 ± 0.88	226.88 ± 0.82	45 ± 0.25	38 ± 0.74
P1 + P2 + P3	50 + 200 + 120	11.22 ± 0.68	0.58 ± 0.35	142.12 ± 0.75	30 ± 0.43	30 ± 0.92
P3	120	56.41 ± 1.02	5.60 ± 0.42	285.57 ± 1.10	35 ± 0.36	35 ± 0.57

* Results expressed as percentage (%) over the total content (relative content). Values are the mean ± SD of two determinations. P1 corresponded to phase 1 (PLE water), P2 to phase 2 (PLE water) and P3 to phase 3 (PLE ethanol).

**Table 3:** Chemical composition of the extracts obtained by the inversion of the sequential process. Compound quantification in spectrophotometer for proteins, carbohydrates, and carotenoids.

Phase	Solvent	Temperature (°C)	Carbohydrates (mg mL^−1^)	Proteins (mg mL^−1^)	Carotenoids (mg mL^−1^)
P1.I	Ethanol	120	56.41 ± 1.02 ^a^	5.60 ± 0.42 ^a^	285.57 ± 1.10 ^a^
P2.I	Water	50	5.51 ± 0.26 ^b^	0 ^b^	9.45 ± 0.85 ^b^
P3.I	Water	100	0.20 ± 0.05 ^c^	0 ^b^	7.45 ± 0.16 ^c^
P4.I	Water	120	0 ^c^	0 ^b^	9.32 ± 0.58 ^d^
P5.I	Water	120	0 ^c^	0 ^b^	2.23 ± 0.17 ^e^

Values are the mean ± SD of two determinations. P1.I corresponded to phase 1 of the inverted process (PLE ethanol), P2.I to phase 2 (PLE water) and P3.I to phase 3 (PLE water). P4.I and P5.I correspond with two additional washes with water; ^a–e^ represent significant differences among values of the same column.

**Table 4:** Compound quantification employing spectrophotometer and HPLC-DAD for carotenoids lutein and astaxanthin.

Phase	Lutein (mg mL^−1^)	Astaxanthin (mg mL^−1^)	Carotenoids (mg mL^−1^)
P1.I	123.84 ± 0.63 ^a^	0.18 ± 0.01 ^a^	285.57 ± 1.10 ^a^
P2.I	123.21 ± 0.25 ^a^	0.90 ± 0.12 ^b^	129.45 ± 0.85 ^b^
P3.I	94.51 ± 0.14 ^b^	1.11 ± 0.05 ^c^	97.45 ± 0.16 ^c^
P4.I	0 ^c^	0 ^d^	9.32 ± 0.58 ^d^
P5.I	0 ^c^	0 ^d^	2.23 ± 0.17 ^e^

Values are the mean ± SD of two determinations. P1.I corresponded to phase 1 of the inverted process (PLE ethanol), P2.I to phase 2 (PLE water) and P3.I to phase 3 (PLE water). P4.I and P5.I correspond with two additional washes with water; ^a–e^ represent significant differences among values of the same column.

## Data Availability

All data supporting the findings of this study are included within the article.

## Abbreviations

The following abbreviations are used in this manuscript:

TLC	Thin-layer chromatography
EPA	Eicosapentaenoic acid
PLE	Pressurized liquid extraction
EAE	Enzymatic-assisted extraction
GLs	Glycolipids
FA	Fatty acid
PUFAs	Polyunsaturated fatty acids
SWE	Subcritical water extraction
GC-MS	Gas chromatography–mass spectrometry
HPLC	High-performance liquid chromatography
BSA	Bovine serum albumine
